# Clinical Characteristics and Outcomes of Patients with Primary Lung Adenocarcinoma Harboring *ALK* Rearrangements Detected by FISH, IHC, and RT-PCR

**DOI:** 10.1371/journal.pone.0101551

**Published:** 2014-07-03

**Authors:** Jinghui Wang, Yiran Cai, Yujie Dong, Jingying Nong, Lijuan Zhou, Guimei Liu, Dan Su, Xi Li, Shafei Wu, Xuejing Chen, Na Qin, Xuan Zeng, Haiqing Zhang, Zongde Zhang, Shucai Zhang

**Affiliations:** 1 Department of Medical Oncology, Beijing Chest Hospital, Capital Medical University, Beijing Tuberculosis and Thoracic Tumor Research Institute, Beijing, China; 2 Department of Pathology, Beijing Chest Hospital, Capital Medical University, Beijing Tuberculosis and Thoracic Tumor Research Institute, Beijing, China; 3 Department of Radiotherapy, Beijing Chest Hospital, Capital Medical University, Beijing Tuberculosis and Thoracic Tumor Research Institute, Beijing, China; 4 Department of Pathology, Peking Union Medical College Hospital, Peking Union Medical College & Chinese Academy of Medical Sciences, Beijing, China; 5 Beijing Key Laboratory of Drug Resistance Tuberculosis Research, Beijing Chest Hospital, Capital Medical University, Beijing Tuberculosis and Thoracic Tumor Research Institute, Beijing, China; Boston University Goldman School of Dental Medicine, United States of America

## Abstract

*EML4*-*ALK* is a new driver gene of non-small cell lung cancer and a target of crizotinib. The objectives of this study were to determine the frequency of *ALK* rearrangements in a large cohort of patients with primary lung adenocarcinoma and to analyze the association of *ALK* rearrangements with clinicopathological characteristics and clinical outcomes. The roles of fluorescence in situ hybridization (FISH), Ventana immunohistochemistry (IHC), and reverse transcriptase polymerase chain reaction (RT-PCR) in the detection of *ALK* rearrangements were evaluated. The *ALK* rearrangement was detected in 430 specimens from individual patients with primary lung adenocarcinoma using FISH and Ventana IHC based on tissue microarrays. The *EGFR* status was detected in all of the specimens through DNA sequencing. An RT-PCR was performed on 200 of the specimens and confirmed by sequencing. Of the 430 patients, 46 (10.7%) harbored *ALK* rearrangements. The *ALK* rearrangements were associated with a younger age and the *EGFR* wild type in comparison with *ALK-*negative patients. The sensitivity and specificity of the Ventana IHC were 100% and 98.2%, respectively, and the concordance rate between the FISH and the Ventana IHC was 98.4%. The sensitivity and specificity of RT-PCR were 95.5% and 87.0%, respectively, and the concordance rate between the FISH and the RT-PCR was 89.0%. The Cox analysis indicated that an early stage and *EGFR-*activating mutations were independently associated with a longer OS. This study demonstrated that *ALK* rearrangements are associated with a younger age and the *EGFR* wild type rather than with other clinicopathological factors. Although the FISH and Ventana IHC have better concordance, and RT-PCR is a more sensitive method and can identify different variants or partners, the IHC and RT-PCR need to be further evaluated in clinical trials to identify their roles in guiding patients’ targeted therapy using crizotinib.

## Introduction

The Echinoderm microtubule-associated protein-like 4 and the anaplastic lymphoma kinase (*EML4-ALK*) fusion genes were discovered in non-small cell lung cancer (NSCLC) in 2007 [1]. The *EML4-ALK* gene is a fusion gene from a chromosome rearrangement between the N-terminal portion of the echinoderm microtubule associated protein-like 4 (*EML4*) gene and the tyrosine kinase (TK) domain of the anaplastic lymphoma kinase (*ALK*) gene, both located on the short arm of chromosome 2, leading to a chimeric oncoprotein with constitutive TK activity and oncogenic transforming activity. Multiple *EML4-ALK* fusion variants have been identified. The truncations of *EML4* may occur at different exons (2, 6, 13, 14, 15, 17, 18 and 20), and the TK domain of the *ALK* gene begins in exon 20 [2]–[5]. In addition to *EML4-ALK*, other *ALK* fusions have also been reported in lung cancer, including *TFG-ALK* [6], *KIF5B-ALK* [7] and *KLC1-ALK* [8]. Crizotinib, an orally available small-molecule tyrosine kinase inhibitor (TKI) targeting ALK, ROS1 and MET, is highly effective in patients with *ALK*-positive NSCLC with an objective response rate of approximately 60% [9]. The U.S. Food and Drug Administration (FDA), the European Medicines Evaluation Agency (EMEA), and the China Food and Drug Administration (CFDA) have granted full approval to crizotinib for the treatment of patients with *ALK*-positive NSCLC.

The incidence of the *ALK* translocation in NSCLC has been reported to be approximately 3–13% in the Western and Chinese populations [10]–[17]. The *ALK* fusion genes appear to be more common in younger patients, patients who were never or light smokers, and patients with adenocarcinoma [17]. Some studies have reported that *ALK* rearrangements were not associated with smoking status [11], [14], [15]. In general, the association between the *ALK* rearrangements and the patient demographics varied depending on the population studied and the screening methods used.

Currently, the three primary methods of detecting *ALK* rearrangements are fluorescent in situ hybridization (FISH), immunohistochemistry (IHC), and the reverse transcriptase polymerase chain reaction (RT-PCR). Each of these individual methods has both advantages and disadvantages. FISH has been considered the gold standard method for detecting *ALK* rearrangements, as it can detect rearrangements irrespective of the *EML4-ALK* gene fusion variants and other fusion partners. However, FISH is expensive, generally requires specialized technical resources and expertise and thus cannot be applied in all pathological laboratories and is unavailable for screening in daily practice. Several antibodies have been investigated for detecting *ALK* rearrangements. Because the expression level of the fusion protein is lower in NSCLC than in anaplastic lymphoma, the development of routine IHC has been problematic [18]–[20]. IHC requires the standardization of reagents and protocols across pathology laboratories. The Ventana ALK assay is a new method of detecting *ALK* rearrangements that uses D5F3 antibody and relies on the tyramide amplification technique bound to the Ventana automated BenchMark XT for high sensitivity. Several studies have demonstrated that there is a high concordance between the Ventana IHC and the FISH [21], [22]. The RT-PCR is a more sensitive and specific method that can identify variants of the *ALK* rearrangements, but all possible *ALK* translocations with *EML4* and the other fusion partners must be accounted for in the primer design to detect them Therefore, detecting *ALK* rearrangements continues to be challenging. Only FISH using a break-apart probe kit (Vysis LSI ALK Dual Color, break-apart rearrangement probe; Abbott Molecular, Abbott Park, IL) is approved by the FDA for the treatment of proven *ALK*-positive NSCLC. However, other detection methods may be approved in the future.

In this study, the *ALK* rearrangements in patients with primary lung adenocarcinoma were investigated, and results obtained through FISH, Ventana IHC, and RT-PCR were compared. The frequency of the *ALK* rearrangements in a group of randomly selected hospitalized patients from Beijing, China, was determined, and its association with clinicopathological characteristics and outcomes were further explored.

## Materials and Methods

### Patients

This study enrolled patients were hospitalized between 2005 and 2013 at Beijing Chest Hospital, Beijing, China. Their clinicopathological data included age, gender, smoking status, tumor, node, metastases (TNM) stage, treatment history, and follow-up. All patients had sufficient tissue for a tissue microarray. Non-smokers were defined as patients who had smoked <100 cigarettes in their lifetime. The TNM staging was reviewed according to the 7^th^ edition of the American Joint Committee for Cancer (AJCC) staging system [23]. The histological subtypes of the adenocarcinomas were classified according to the new International Association for the Study of Lung Cancer/American Thoracic Society/European Respiratory Society (IASLC/ATS/ERS) multidisciplinary classification of lung adenocarcinoma [24]. The Response Evaluation Criteria in Solid Tumors (RECIST) [25] was used, including complete response (CR), partial complete (PR), stable disease (SD), and progression of disease (PD). The overall response rate (ORR) contains the CR and the PR. The progression-free survival (PFS) time was measured from the first date of treatment until the date of the first documented disease progression or until the date of death for any reason in the absence of disease progression. The overall survival (OS) in this study was measured from the date of the first operation for patients who underwent surgery or of first-line anti-tumor therapy for advanced patients until the date of death for any reason. The present study was conducted according to the principles of the Declaration of Helsinki and approved by the ethical committees of Beijing Chest Hospital. All of these patients assigned written informed consent, and the ethics committees approved the consent procedure.

### Specimen preparation

A slide was cut from the formalin-fixed, paraffin-embedded (FFPE) blocks of selected patients with primary lung adenocarcinoma for hematoxylin and eosin staining. The samples containing more than 75% tumor cells were enrolled. Other three- to five 5-µm-thick slides were cut from the blocks and placed into two Eppendorf tubes for the DNA extraction and the RNA extraction. Three cores of the tissue, each with a diameter of 2 mm from the tumor areas marked by the pathologists, were patched from the block for each patient and were used for tissue microarrays (TMA). The *EGFR* status of each of the patients was identified by the DNA sequencing method. Serial sections of the TMAs were cut, and hematoxylin and eosin staining, FISH, and IHC were performed.

### Detection of *ALK* rearrangements

FISH was performed on the 4-µm-thick slides of FFPE TMA using the Vysis ALK Break Apart FISH Probe Kit (Vysis LSI ALK Dual Color, break-apart rearrangement probe; Abbott Molecular, Abbott Park, IL) according to the manufacturer’s instructions. At least 100 representative tumor cells were counted, and the occurrence of an *ALK* gene rearrangement was concluded if ≥15% of the tumor cells showed a split red and green signal and/or an isolated (single) red signal. Otherwise, the specimen was classified as *ALK* FISH negative. The results obtained by FISH were analyzed using an Olympus fluorescence microscope equipped with orange, green, and 4′, 6-diamidino-2-phenylindole filters. Images were captured using the Video Test Image Analysis System.

IHC was performed on the 4-µm-thick slides of FFPE TMA on a Benchmark XT stainer. The pre-diluted Ventana anti-ALK (D5F3) rabbit monoclonal primary antibody (Cell Signal Technology, U.S.) was applied, and the Optiview DAB IHC detection kit and the Optiview Amplification kits were used according to the manufacturers’ instructions. Each patient sample included a matched rabbit monoclonal IgG negative control. The scoring algorithm for the ALK IHC was that the presence of strong granular cytoplasmic staining in the tumor cells (any percentage of positive tumor cells) were concluded to be positive for ALK, while the absence of strong cytoplasmic staining in the tumor cells indicated that they were negative for ALK.

The *ALK* rearrangements were tested by RT-PCR using an AmoyDx ALK Fusion Gene Detection Kit (Amoy Diagnostics, Xiamen, China). The positive PCR products were verified by sequencing on an ABI 3500dx at Amoy Diagnostics, China. The primers sequences used on sequencing are followed: EML4-ALK variant 1: F: GGAGCAAAACTACTGTAGAGCCCA, M13R-R: AGCGGATAACAATTTCACACAGGACTTGCAGCTCCTGGTGCT; EML4-ALK variant 2: F: ACAAGTATATAATGTCTAACTCGGGAG, M13R-R: AGCGGATAACAATTTCACACAGGACTTGCAGCTCCTGGTGCT; EML4-ALK variant 3a/b: F: ACTGCAGACAAGCATAAAGATGTC, M13R-R: AGCGGATAACAATTTCACACAGGACTTGCAGCTCCTGGTGCT; KIF5B-ALK: F: AGTAGATCGCATAAAGGAAGCAGTC, M13R-R: AGCGGATAACAATTTCACACAGGACTTGCAGCTCCTGGTGCT.

### Statistical analysis

Fisher’s exact test was used to examine the association between the *ALK* rearrangements and the clinicopathological factors. The continuous data were analyzed by the Kruskal-Wallis test. The ORR was compared by Fisher’s exact test between the different groups. The Kaplan-Meier method was used to estimate the PFS and the OS and the log-rank test was used to analyze the PFS or OS between the different groups. The Cox proportional hazards regression model was used to identify independent factors of OS. All of the statistical tests were performed using the SPSS 16.0 software (SPSS Inc., Chicago, IL). The *P* values were 2 tailed for all of the tests. Statistical significance was set at *P*<0.05.

## Results

### Patients

A total of 430 patients with primary lung adenocarcinoma were available for analysis. The clinicopathological characteristics of all of the patients with lung adenocarcinoma are listed in Table 1.

**Table 1:** Characteristics of all patients.

Characteristics	N (%)
Age (year)	
Range	23–82
Median	57
Gender	
Male	229 (53.3)
Female	201 (46.7)
Smoking status	
Non-smoking	263 (61.2)
Smoking	167 (38.8)
Stage	
I	78 (18.1)
II	28 (6.5)
IIIA	114 (26.5)
IIIB+IV	201 (46.7)
Stage unknown	9 (2.1)
Histologic subtype	
Lepidic predominant	12 (2.8)
Acinar predominant	236 (54.9)
Papillary predominant	101 (23.5)
Micropapillary predominant	20 (4.7)
Solid predominant with mucin production	51 (11.9)
Invasive mucinous adenocarcinoma	7 (1.6)
Colloid variant	3 (0.7)
*EGFR* status	
Mutation	186 (43.3)
Exon 18 mutation	9 (2.1)
Exon19 mutation	94 (21.9)
Exon 20 mutation	3 (0.7)
Exon 21 mutation	77 (17.9)
Multiple mutation	3 (0.7)
Wild type	244 (56.7)

### *ALK* rearrangements

Forty-six (10.7%) of the patients were identified as *ALK-*positive by FISH, of which one patient had a co-mutation with the *ALK* rearrangement and *EGFR* mutation (L858R), and 199 (46.3%) had the wild type for both the *ALK* and the *EGFR* (defined as WT/WT). The association between the genotypes and clinicopathological characteristics are shown in Table 2.

**Table 2:** Association of different genotypes with clinicopathological characteristics in 430 patients (*ALK* rearrangement results based on FISH detection).

	*ALK* rearrangement (n = 46)#		*EGFR* mutation (n = 186)#		WT/WT (n = 199)		*P (ALK* vs. *EGFR*)	*P (ALK vs*. WT/WT)
	n	%	n	%	n	%		
Age (years old)								
Median	52		57		60		<0.001	<0.001
Range	23–69		28–79		26–82			
Gender								
Male	25	54.3	77	41.4	128	64.3	0.136	0.238
Female	21	45.7	109	58.6	71	35.7		
Smoking status								
Non-smoking	29	63.0	136	73.1	98	49.2	0.204	0.103
Smoking	17	37.0	50	26.9	101	50.8		
Stage								
I	6	13.0	41	22.0	31	15.6	0.129	0.129
II	0	0.0	9	4.8	19	9.5		
IIIA	12	26.1	53	28.5	50	25.1		
IIIB+IV	28	60.9	78	41.9	95	47.7		
Unknown stage	0	0.0	5	2.7	4	2.0		
Histologic subtype*								
Lepidic predominant	0	0.0	7	3.8	5	2.5	<0.001	0.074
Acinar predominant	27	58.7	99	53.2	110	55.3		
Papillary predominant	4	8.7	58	31.2	39	19.6		
Micropapillary predominant	3	6.5	7	3.8	10	5.0		
Solid predominant	11	23.9	15	8.1	26	13.1		
Invasive mucinous adenocarcinoma	0	0.0	0	0.0	7	3.5		
Colloid variant	1	2.2	0	0.0	2	1.0		

# One patient had a co-mutation of *EGFR* and *ALK*.

*P value was analysis on the frequency of *ALK* rearrangement among acinar predominant subtype, papillary predominant subtype, and solid predominant subtypes because of small samples of other subtypes.

**Figure 1:**
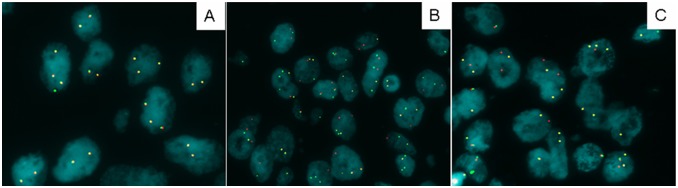
Detection of *ALK* rearrangements using Fluorescent in situ hybridization (FISH) (1000×). (A) An *ALK-*negative case. (B) An *ALK-*positive case with split signal pattern. (C) An *ALK-*positive case with isolated red signal pattern.

Compared with the patients with the *EGFR* mutations, the *ALK-*positive patients were significantly younger than the patients harboring the *EGFR* mutations, with a median age of 52 compared with 57 (*P*<0.001), respectively. The incidence of *ALK* rearrangements in the solid subtype was significantly higher than in the acinar subtype and the papillary subtype (*P* = 0.044, *P*<0.001); the incidence of the *ALK* rearrangements in the acinar subtype was significantly higher than in the papillary subtype (*P* = 0.011). There were no differences in the incidence of the *ALK* rearrangements and *EGFR* mutations for gender, smoking status, or stage. Compared with the WT/WT patients, the *ALK-*positive patients were significantly younger than the patients harboring the WT/WT genotype, with a median age of 52 compared with 60 (*P*<0.001), respectively. There were no differences in the gender, smoking status, stage, or histological subtype between the *ALK*-positive and the WT/WT patients. The incidence of the *ALK* rearrangements in the patients harboring the *EGFR* wild type was significantly higher than in the *EGFR* mutations (45/244, 18.4% versus 1/186, 0.5%, *P*<0.001). Images of the results of the FISH are shown in Figure 1.

**Figure 2:**
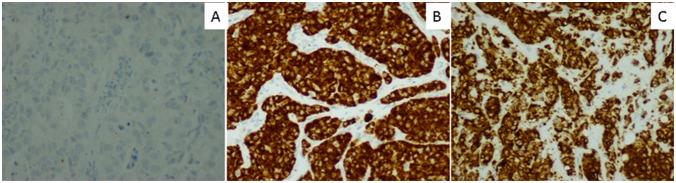
Detection of *ALK* rearrangements using Ventana immunohistochemistry (IHC) (200×). (A) An *ALK-*negative case without cytoplasmic staining. (B) An *EML4-ALK-*positive case with strong granular cytoplasmic staining. (C) A *KIF5B-ALK-*positive case identified by RT-PCR with strong granular cytoplasmic staining.

The IHC showed that 53 (12.3%) patients were *ALK-*positive with a strong granular cytoplasmic staining in the tumor cells (Figure 2). The incidence of the *ALK* rearrangements by the IHC in patients harboring the *EGFR* wild type was significantly higher than in the patients with the *EGFR* mutations (52/244, 21.3%, 1/186, 0.5%; *P*<0.001).

RT-PCR was performed for 200 patients, including 46 with *ALK-*positive and 154 with *ALK-*negative results detected by FISH. Sixty-four patients were positive for the *ALK* rearrangements (32.0%). All of the *ALK-*positive specimens were confirmed by direct sequencing. Among the *ALK-*positive patients, 62 (96.9%) had *EML4-ALK* fusion genes, and 2 (3.1%) had *KIF5B-ALK* fusion genes in which exon 24 of the *KIF5B* and exon 20 of the *ALK* were jointed. The variant fusion types were identified from the 62 patients with the *EML4-ALK* fusion genes. These included variant 1 (E13;A20) in 24 patients (38.7%), variant 2 (E20;A20) in eight patients (12.9%), and variant 3a/b (E6a/b;A20) in 30 patients (48.4%). The two patients harboring *KIF5B-ALK* fusion gene had no *EGFR* mutations and belonged to the acinar subtype. Both of them were non-smoking males, one of whom was 48 years old with stage IIIA and died 34.5 months after surgery and the other of whom was 55 years old with stage IV and died 6 months after diagnosis.

Of the 200 patients detected by RT-PCR, 84 patients had *EGFR* mutations and 116 patients had the wild type. The incidence of the *ALK* rearrangements in the patients harboring the *EGFR* wild type was significantly higher than in the patients with the *EGFR* mutations (53/116, 45.7%, 11/84, 13.1%; *P*<0.001). The details of the 11 patients who had co-mutations with the *ALK* rearrangement and the *EGFR* mutation are shown in Table 3.

**Table 3:** Details of 11 patients with a co-mutation of *EGFR* and *ALK* by RT-PCR.

Case	Age	Gender	Smoking status	Stage	Histologic subtype	*EGFR* Status	Mutation typeof *EGFR*	FISH	Ventana IHC	RT-PCR	Variants or partners
1	57	Female	Non-smoking	IIIA	Acinar predominant	Mutation	19 deletion	Negative	Negative	Positive	v3a/b
2	55	Female	Non-smoking	IV	Acinar predominant	Mutation	19 deletion	Negative	Negative	Positive	v3a/b
3	49	Male	Smoking	IV	Acinar predominant	Mutation	19 deletion	Negative	Negative	Positive	v1
4	45	Male	Non-smoking	IV	Acinar predominant	Mutation	19 deletion	Negative	Negative	Positive	v1
5	34	Female	Non-smoking	IV	Acinar predominant	Mutation	19 deletion	Negative	Negative	Positive	v3a/b
6	34	Female	Non-smoking	I	Micropapillary predominant	Mutation	19 deletion	Negative	Negative	Positive	v1
7	46	Male	Smoking	I	Papillary predominant	Mutation	19 deletion	Negative	Negative	Positive	v1
8	58	Male	Smoking	IIIA	Solid predominant	Mutation	L858R	Positive	Positive	Positive	v3a/b
9	63	Female	Non-smoking	I	Papillary predominant	Mutation	19 deletion	Negative	Negative	Positive	v3a/b
10	56	Female	Non-smoking	IV	Acinar predominant	Mutation	19 deletion	Negative	Negative	Positive	v3a/b
11	60	Male	Smoking	I	Papillary predominant	Mutation	19 deletion	Negative	Negative	Positive	v1

### Comparison of the *ALK* rearrangements detection among FISH, Ventana IHC, and RT-PCR

In the correlation analysis of these three methods, FISH was considered to be the gold standard method. Of the 430 patients detected by FISH and IHC, 46 patients were both FISH and IHC positive, 7 patients were IHC positive/FISH negative, and 377 patients were both FISH and IHC negative. The IHC sensitivity and specificity were 100% and 98.2%, respectively. The concordance rate between the FISH and the IHC for detecting the *ALK* rearrangements was 98.4%.

Of the 200 patients detected by FISH, IHC and RT-PCR, 44 of the 46 FISH positive patients were also IHC positive/RT-PCR positive. Two of the FISH positive/IHC positive patients were negative on the RT-PCR. Of the 7 FISH negative/IHC positive patients, 6 patients were positive on the RT-PCR, and 1 patient was RT-PCR negative. Six of the 20 patients who were FISH negative/RT-PCR positive were IHC positive, and 14 patients were IHC negative One hundred and thirty-three patients were FISH/IHC/RT-PCR negative. Two patients harboring the *KIF5B-ALK* fusion gene were also determined to be *ALK* positive by FISH and IHC. The sensitivity and specificity of the RT-PCR were 95.7% and 87.0%, respectively. The concordance rate of the FISH and the RT-PCR was 89.0% (Table 4).

**Table 4:** Comparison of FISH and IHC in 430 patients and comparison of FISH and RT-PCR on detecting *ALK* rearrangements among 200 patients.

		FISH		Total	Sensitivity	Specificity	Concordance rate
		Positive	Negative				
Ventana IHC	Positive	46	7	53	100%	98.2%	98.4%
	Negative	0	377	377			
	Total	46	384	430			
RT-PCR	Positive	44	20	64	95.7%	87.0%	89.0%
	Negative	2	134	136			
	Total	46	154	200			

### The outcomes

**Figure 3:**
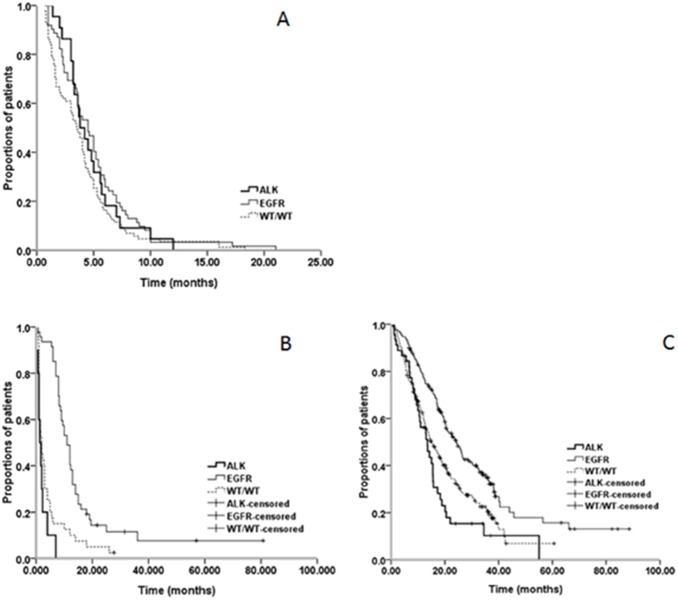
Progression-free survival (PFS) and overall survival (OS) among *ALK-*positive patients, patients who have *EGFR* activating mutations and wild type both *ALK* and *EGFR* (WT/WT). (A) PFS for patients receiving the first-line chemotherapy harboring *ALK* rearrangements, *EGFR* activating mutations, and WT/WT. (B) PFS for patients receiving EGFR TKIs harboring *ALK* rearrangements, *EGFR* activating mutations, and WT/WT. (C) OS for patients harboring *ALK* rearrangements, *EGFR* activating mutations, and WT/WT.

Of the 430 patients, 216 patients with recurrent or advanced disease who received systemic treatment were available for an analysis of its efficacy. For an analysis of the efficacy, the patients were divided into three groups consisting of the *ALK* rearrangements, the *EGFR* activating mutations (exon 19 deletions and exon 21 mutation), and the WT/WT for both the *ALK* rearrangements and *EGFR* wild types. Of the 216 patients, 171 patients with recurrences or advanced patients received chemotherapy as the first- line treatment and 45 patients received TKIs as the first- line treatment. Among the 171 patients, 22 had *ALK* rearrangements, 62 had *EGFR* activating mutations, and 87 had the WT/WT. The best response to the first-line chemotherapy was analyzed. The ORR of the chemotherapy for patients with *ALK* rearrangements, *EGFR* activating mutations, and WT/WT were 31.8%, 37.1%, and 25.3%, respectively. The PFS of the first-line chemotherapy for patients harboring *ALK* rearrangements, *EGFR* activating mutations, and WT/WT were 3.8 months, 4.5 months, and 3.5 months, respectively. There were no significant differences in the ORR and PFS among the three groups in the first-line chemotherapy (Figure 3A). Among the 171 patients, 46 patients received pemetrexed combined platinum or pemetrexed monotherapy. Of the 46 patients, three patients were *ALK*-positive, of which patients received pemetrexed treatment as the first line. The response rate and PFS were SD and 7.3 months and PR and 12 months, respectively. The third *ALK-*positive patient received pemetrexed treatment in the second line had SD and the PFS was 6 months. A statistical analysis was not conducted due to the small sample size of the patients received pemetrexed.

Of the 216 patients, 97 patients who received the EGFR TKIs treatment were available for an analysis of the response, including 45 patients in the first line, 43 patients in the second line, and nine patients in the third line. Among the patients receiving the EGFR TKIs, 10 had *ALK* rearrangements, 47 had *EGFR* activating mutations, and 40 had the WT/WT. The best response to the EGFR TKIs in all of the lines was analyzed. The ORR of EGFR TKIs for patients harboring *ALK* rearrangements, *EGFR* activating mutations, and the WT/WT were 0.0%, 68.1%, and 12.5%, respectively. The ORR for the patients harboring the *EGFR* activating mutations was significantly higher than for the patients harboring the *ALK* rearrangements and the WT/WT (*P*<0.001, *P*<0.001, respectively). The PFS for the patients harboring the *ALK* rearrangements, *EGFR* activating mutations, and WT/WT were 1.3 months, 11.0 months, and 2.0 months, respectively. The PFS for the patients harboring the *EGFR* activating mutations was significantly higher than for the patients harboring the *ALK* rearrangements or WT/WT (*P*<0.001, *P*<0.001, respectively) (Figure 3B). No significant differences in the ORR and PFS between the *ALK* rearrangements and the WT/WT group were observed. The efficacy for the patients with different genotypes receiving chemotherapy or EGFR TKIs is shown in Table 5.

**Table 5:** Treatment response and PFS according to different genotypes in patients with recurrent or advanced diseases.

	n	*ALK* rearrangement	*EGFR* activating mutation	WT/WT	*P (ALK* vs. *EGFR*)	*P (ALK* vs. WT/WT)	*P (EGFR* vs. WT/WT)
No. of patients evaluated in first line chemotherapy	171	22	62	87			
CR		0 (0.0)	0 (0.0)	0 (0.0)			
PR		7 (31.8)	23 (37.1)	22 (25.3)			
SD		12 (54.5)	31 (50.0)	36 (41.4)			
PD		3 (13.6)	8 (12.9)	29 (33.3)			
ORR		7 (31.8)	23 (37.1)	22 (25.3)	0.797	0.592	0.086
PFS, month (95% CI)		3.8 (2.766–4.834)	4.5 (3.518–5.482)	3.5 (2.867–4.133)	0.694	0.407	0.041
No. of patients evaluated in any-line TKIs therapy	97	10	47	40			
CR		0 (0.0)	2 (4.3)	0 (0.0)			
PR		0 (0.0)	30 (63.8)	5 (12.5)			
SD		3 (30.0)	12 (25.5)	12 (30.0)			
PD		7 (70.0)	3 (6.4)	23 (57.5)			
ORR		0 (0.0)	32 (68.1)	5 (12.5)	<0.001	0.569	<0.001
PFS, month (95% CI)		1.3 (0.215–2.385)	11.0 (8.901–13.099)	2.0 (1.229–2.771)	<0.001	0.169	<0.001

The latest follow-up was performed on 30^th^, September 2013. Of the 430 patients investigated, 299 patients had died, 94 patients were still alive, and 37 patients failed to follow-up. The overall survival was analyzed in the patients with early stage and advanced disease. The patients who were still alive or failed to follow-up were regarded as censors in the statistical analysis.

Of the 299 patients, the median OS was 18.7 months (95% CI 16.892–20.508). The OS for the patients who had *ALK* rearrangements, *EGFR* activating mutations, and WT/WT were 13.5 (95% CI 9.599–17.401), 24.2 (95% CI 19.869–28.531), and 15.0 (95% CI 12.206–17.794) months, respectively. The OS for the patients harboring *EGFR* activating mutations was significantly longer than for the patients harboring *ALK* rearrangements and WT/WT (*P*<0.001, *P*<0.001). There was no significant difference in the OS between the *ALK-*positive patients and the WT/WT patients *(P* = 0.123) (Figure 3C).

Factors including the age, gender, smoking status, stage, histological subtypes, *EGFR* status, *ALK* status, and EGFR TKIs treatments were analyzed for their association with the OS. Due to the small number of patients with unknown stage, patients with lepidic, micropapillary, invasive mucinous adenocarcinoma and colloid variants were not enrolled in the analysis. The univariate analysis showed that the stage (31.2 months for stage I-IIIA vs. 10.7 months for stage IIIB-IV, *P*<0.001), histological subtypes (15.3 months for the acinar subtype, 24.2 months for the papillary subtype, 17.3 months for the solid subtype, *P*<0.001), *EGFR* status (24.2 months for the activating mutations vs. 14.3 months for the wild type, *P*<0.001), and *ALK* status (20.0 months for the *ALK*-negative vs. 13.0 months for the *ALK-*positive, *P = *0.001) were associated with the OS. Age (18.9 months for <57 years old vs. 18.0 months for ≥57 years old, *P* = 0.301), gender (18.2 months for male vs. 18.9 months for female, *P* = 0.632), smoking status (18.9 months for non-smoking vs. 18.2 months for smoking, *P* = 0.612), and EGFR TKIs treatment (20.5 months for EGFR TKIs treatment vs. 18.0 months without TKIs treatment, *P* = 0.589) were not associated with the OS. The multivariate analysis was performed using a Cox regression model. The Cox analysis showed that an early stage (*P*<0.001, HR 3.707, 95% CI 2.841–4.836) and *EGFR* activating mutations (*P*<0.001, HR 1.697, 95% CI 1.306–2.205) were independently associated with a longer OS, whereas the histological subtypes (*P* = 0.129, HR 0.869, 95% CI 0.725–1.042) and the *ALK* status were not independent predictors of OS (*P = *0.212, HR 0.786, 95% CI 0.539–1.147) (Table 6).

**Table 6:** Univariate and multivariate analysis for overall survival.

Variables	OS (month)	Univariate		Multivariate	
		*P*	95% CI	*P*	HR (95% CI)
Age (years old)					
<57	18.9	0.301	16.196–21.604		
≥57	18.0		15.514–20.486		
Gender					
Male	18.2	0.632	15.113–21.287		
Female	18.9		16.723–21.077		
Smoking status					
Non-smoking	18.9	0.612	16.444–21.356		
Smoking	18.2		15.522–20.878		
Stage					
I+II+IIIA	31.2	<0.001	25.134–37.266	<0.001	3.707 (2.841–4.836)
IIIB+IV	10.7		9.231–12.169		
Histologic subtypes					
Acinar	15.3	<0.001	12.622–17.978	0.129	0.869 (0.725–1.042)
Papillary	24.2		16.628–31.772		
Solid	17.3		14.131–20.469		
*EGFR* status					
Activating mutation	24.2	<0.001	19.923–28.477	<0.001	1.697 (1.306–2.205)
Wild type	14.3		12.350–16.250		
*ALK* status					
Positive	13.0	0.001	9.484–16.516	0.212	0.786 (0.539–1.147)
Negative	20.0		17.627–22.373		
EGFR TKIs treatment					
With	20.5	0.589	16.388–24.612		
Without	18.0		15.585–20.415		

## Discussion

The *ALK* rearrangements were screened in this study in a large randomly selected cohort of patients with lung adenocarcinoma by FISH, IHC, and RT-PCR. According to the FISH results, *ALK-*positive patients were associated with a younger age and with the *EGFR* wild type rather than with other clinicopathological factors.

The incidence of *ALK* rearrangements was 10.7% (46/430) as obtained by FISH in this study, which was similar to the results previously published [13], [14]. We also found that the *ALK* fusion genes are associated with a younger age and the *EGFR* wild type, which was consistent with previous studies [13], [17], [26]. However, we did not find that the *ALK* rearrangements were associated with non-smoking. This was similar to some of the previous work [11], [13], [16], [26], [27], [28], but differed from others [17], [29], which might be due to the difference in the population studied. Previous studies reported that *ALK* rearrangements were common in signet cell carcinoma and mucinous adenocarcinoma [19]. According to the new classification of lung adenocarcinoma by the IASLC/ATS/ERS, there were no associations between the *ALK* rearrangements and histological subtypes.

Previous studies have shown that the Ventana IHC has a high sensitivity and specificity (>98%), as well as good concordance with FISH [21], [22]. In our study, the Ventana IHC was performed in all of the 430 patients, with a sensitivity and specificity of 100% and 98.2%, respectively. It demonstrated a high concordance rate of 98.4%, which was similar to the results obtained from the above studies. In addition to its high coherence with FISH, Ventana IHC is quicker, less expensive, easier to control, and has a good repeatability, and it has been approved by the EMEA and CFDA as an aid in identifying patients who are eligible for treatment with crizotinib.

RT-PCR is a highly sensitive method demonstrated in this study. It had a positive rate of 32.0% (64/200) for the detection of *ALK* rearrangements in 200 patients, which was the highest compared with 23.0% (46/200) and 26.5% (53/200) detected by FISH and IHC, respectively, which was further confirmed by sequencing. RT-PCR can also identify variants or different fusion partners. In our study, variant 1 (38.7%) and variant 3a/b (48.4%) were the common variants, and variant 2 was less common, which is similar to a previous study reporting that the most common variants were variant 1 (33%) and variant 3a/b (29%) [1]. However, the frequency was different, which may be due to the population studied. The exact frequency of the variants and their clinical significance remain under investigation. Different *EML4-ALK* variants may exhibit different sensitivities to crizotinib [30]. *KIF5B-ALK* was detected in two cases with variants of *KIF5B-ALK* in which exon 24 of *KIF5B* and exon 20 of *ALK* were joined. FISH and IHC also detected the *KIF5B-ALK* fusion gene in these two patients, which demonstrated that both methods are independent of fusion partners. The RNA quantity and quality are critical for the detection of the *ALK* rearrangement using RT-PCR. RNA degradation of the FFPE may occur, which induces false negative results. Two patients who were FISH and IHC positive but were negative on the RT-PCR may be explained by the RNA degradation in the FFPE tissue samples. The other explanation may be related to RT-PCR, which can only detect the known rearrangements. As RT-PCR is a PCR-based method, the sample contamination issue should be considered in the process of cutting the slides from the tissue and in the preparation of the PCR reaction mix. The sequencing of the PCR product following the RT-PCR procedure is a good method to ensure the accuracy of the result.

In this study, the RT-PCR revealed a sensitivity and specificity of 95.7% and 87.0%, respectively, and a concordance rate of 89.0% between the FISH and the RT-PCR, which was slightly lower than in a previous study [21]. We had a higher frequency of cases that they were RT-PCR positive but FISH negative. This may be related to the differences in the sample types collected from the patients enrolled. The majority of the samples in this study were from resected tumors or lymph nodes rather than a small biopsy. These types of samples enabled the acquisition of a higher quality and quantity of RNA and improved the positive rates. There are also several reasons for the discordance between the FISH and RT-PCR: first, splitting the red and green FISH signals can be extremely subtle, which leads to false-negative results, and a small proportion of atypical results from an ALK FISH interpretation may be discrepant; second, the cross-contamination of RT-PCR samples and non-specific amplification may also cause false positive results. In our study, the positive *ALK* rearrangement observed on the RT-PCR in patients with the *EGFR* wild type was 45.7% (53/116), which was higher than the result of 34% reported in a previous study [27]. The reason for these varied results may be related to the population and to the tissue differences. The correlation between FISH and RT-PCR needs to be demonstrated further in a large sample population.

Using FISH or IHC, one of the 430 (1/186, 0.5%) patients, a 58-year-old male patient with solid subtype had a coexisting of *ALK* rearrangement and *EGFR* mutation (L858R in exon 21). On the RT-PCR, the frequency of the concomitant mutation was 13.1% (11/84), which was similar to the 15.8% of the coexistence of *ALK* and *EGFR* with RT-PCR [31]. The frequency may be due to the high sensitivity of the RT-PCR. In regard to the reasons for the different frequencies of the concomitant mutations on FISH and RT-PCR, some of which are similar to those noted above, may be due to false negatives on FISH or false positives on RT-PCR. In addition, Wallander [32] reported that cases with *EML4*-*ALK* variant 1, as detected by RT-PCR, were hardly detectable by IHC, with either no or faint staining on FISH, as the distance between the two ALK probes was less than two signal distances apart. To date, the benefits of crizotinib for *ALK*-positive patients are available from clinical trials based on the Vysis break-apart FISH assay. However, to examine the ability of these multiple different *ALK* detection assays to predict the benefits of crizotinib, well-designed prospective studies including these assays should be constructed in the future.

In the present study, we also analyzed the outcomes based on the genotypes. No differences in the ORR and the PFS for the first-line chemotherapy were observed in patients who were *ALK-*positive and had *EGFR* activating mutations and the WT/WT. The response rate and PFS of the EGFR TKIs for any lines in the patients harboring *EGFR* activating mutations was significantly higher and longer than in patients who were *ALK-*positive and the WT/WT. These results are quite similar to the studies reported previously [17], [26], [28]. Previous studies reported that *ALK*-positive patients have a significantly longer PFS on pemetrexed [33], [34]. In our cohort, three *ALK-*positive patients received pemetrexed treatment, and the PFS for the three patients was 6, 7.3 and 12 months. Due to the very small sample size, the difference in the response to pemetrexed between the *ALK-*positive and negative patients was not compared.

In the OS analysis, the OS for patients harboring *EGFR* activating mutations was significantly longer than for patients harboring *ALK* rearrangements or WT/WT, and there was no difference in the OS between patients harboring *ALK* rearrangements and WT/WT. These results were also similar to the previous studies [17], [28]. For the analysis of the predictors of OS, the univariate analysis showed that the stage, histological subtypes, *EGFR* status and *ALK* status were associated with the OS. The Cox analysis demonstrated that an early stage and *EGFR* activating mutations were independent factors of longer overall survival. However, the *ALK* status was not an independent factor of OS, which is similar to the finding in a previous study [17].

In conclusion, the *ALK* rearrangement was studied in a large unselected sample collection of lung adenocarcinoma. An occurrence of 10.7% of *ALK* rearrangements was identified, which was associated with a younger age and the *EGFR* wild type. The FISH and the Ventana IHC had a better concordance. RT-PCR is a more sensitive method and can identify different variants or partners. The IHC and RT-PCR need to be further evaluated in clinical trials to identify their roles in guiding patients’ targeted therapy using crizotinib. In an era of targeted therapy, the status of *EGFR* and *ALK* should be identified in patients with lung adenocarcinoma before treatment.
